# Suicidal ideation and behavior in youth in low- and middle-income countries: A brief review of risk factors and implications for prevention

**DOI:** 10.3389/fpsyt.2022.1044354

**Published:** 2022-12-06

**Authors:** Johanne Renaud, Sasha Leigh MacNeil, Lakshmi Vijayakumar, Michel Spodenkiewicz, Sylvanne Daniels, David A. Brent, Gustavo Turecki

**Affiliations:** ^1^McGill Group for Suicide Studies, Douglas Mental Health University Institute, Montréal, QC, Canada; ^2^Department of Psychiatry, Faculty of Medicine and Health Sciences, McGill University, Montréal, QC, Canada; ^3^Department of Psychology, Concordia University, Montréal, QC, Canada; ^4^Sneha Suicide Prevention Centre, Chennai, Tamil Nadu, India; ^5^Pôle de Santé Mentale, CIC-EC 1410, Université et CHU de La Réunion Sainte-Pierre, Saint-Pierre, France; ^6^INSERM UMR-1178 Moods Team CESP Le Kremlin-Bicêtre France, Le Kremlin-Bicêtre, France; ^7^Western Psychiatric Institute and Clinic, University of Pittsburgh Medical Center, Pittsburgh, PA, United States

**Keywords:** suicide, suicidal ideation, suicide attempts, youth, low-and middle-income countries, prevention

## Abstract

Although global rates of suicide have dropped in the last 30 years, youth in low- and middle-income countries (LMICs) continue to be highly represented in suicide statistics yet underrepresented in research. In this review we present the epidemiology of suicide, suicidal ideation, and suicide attempts among youth in LMICs. We also describe population-level (attitudes toward suicide, socioeconomic, and societal factors) and individual-level clinical and psychosocial risk factors, highlighting specific considerations pertaining to youth in LMICs. These specific considerations in risk factors within this population can inform how multi-level prevention strategies may be targeted to meet their specific needs. Prevention and intervention strategies relying on the stepped-care framework focusing on population-, community-, and individual level targets while considering locally- and culturally relevant practices are key in LMICs. In addition, systemic approaches favoring school-based and family-based interventions are important among youth. Cross-culturally adapted multimodal prevention strategies targeting the heterogeneity that exists in healthcare systems, suicide rates, and risk factors in these countries should be accorded a high priority to reduce the burden of suicide among youth in LMICs.

## Introduction

Suicide is an important global public health issue, claiming over 700,000 lives per year (1). Following a number of different actions, including World Health Organization’s (WHO) calls for reductions in suicide mortality (1, 2), global suicide mortality rates have dropped by over one third in the last thirty years, with age-standardized suicide rates of 9.0 per 100,000 in 2019 (3), compared to 16.6 per 100,000 in 1990 (4, 5). While this achievement has its merits, it is equally important to recognize the longstanding challenges to obtaining precise estimates of suicide rates in certain countries and regions (6), or that these declining rates are not consistently seen across all countries and demographics, with some continuing to carry a disproportionately high burden of suicide. This particularly concerns data on child and adolescent suicides for which reliable data are lacking in developing countries (7). Suicide remains a leading cause of death among youth aged 15–29 years old (8). Moreover, reports indicate that 77% of suicides occur in low- and middle-income countries (LMICs), i.e., countries with a gross national income (GNI) per capita of 12,695 USD or less (9), due to the large proportion of the global population living in these countries (5).

Therefore, youth in LMICs may represent a population with a high burden of suicidal ideations and behaviors, presenting with a different declination of risk factors, including a particular psychosocial profile and barriers to accessing care. In this brief review, our first objective consists in reviewing the epidemiology of suicide and suicidal ideation and attempts among young people in LMICs, including adolescents (i.e., individuals aged 10–19 years) and youths (i.e., individuals aged 15–29 years). Secondly, we want to revise specific risk factors for suicidality (at both population- and individual-levels), as they pertain to young people in LMICs. Lastly, we discuss how these risk factors may help inform prevention and intervention strategies among this population that continues to be highly represented in suicide statistics yet underrepresented in research due to practical and logistical obstacles to accessing reliable data.

## Epidemiology

### Suicide mortality

#### Global population

Eighty to 84% of the global population lives in LMICs (10). Out of 140 LMICs, 78 countries do not have a vital registration system and hence most estimates of suicide rates are based on nationally representative or regional reports and modeling algorithms (1). The most recent and reliable data from the WHO report on suicide from 2019 indicate that age-standardized suicide mortality rates are generally lower in LMICs (ranging from 7.28 to 10.07 per 100,000 people) than in high-income countries (HICs; 10.95 per 100,000 population) (11). However, the 11 countries with the highest suicide rates are LMICs, with rates ranging from 87.48 per 100,000 population (Lesotho) to over 20 per 100,000 (Central African Republic, Russia) (3). In addition, global suicide rates among men are higher in HICs than in LMICs across all age groups (19.9 vs. 13.7 per 100,000 people) whereas suicide rates among women are lower in HICs than in LMICs (5.7 vs. 8.7 per 100,000) (12). Despite these global trends, suicide rates among LMICs vary greatly, with other countries reporting rates under 3 per 100,000 (Lebanon, Syria, Kuwait, and Jamaica) (3). China and India, with respective decreases in suicide rates of 64 and 32.7% from 1990 to 2016, have been key drivers of the recent global decline in suicide (4) since they are both very populous countries in which major gains have had a noticeable impact on global statistics. Yet, these countries together still contribute 44.2% of global suicides despite representing approximately 36% of the world population (4, 13). Other countries (e.g., Brazil) have not seen the same drop in suicide in the last decade, with instead significant increases in rates since 2000 (14).

#### Adolescents and youth

**FIGURE 1:**
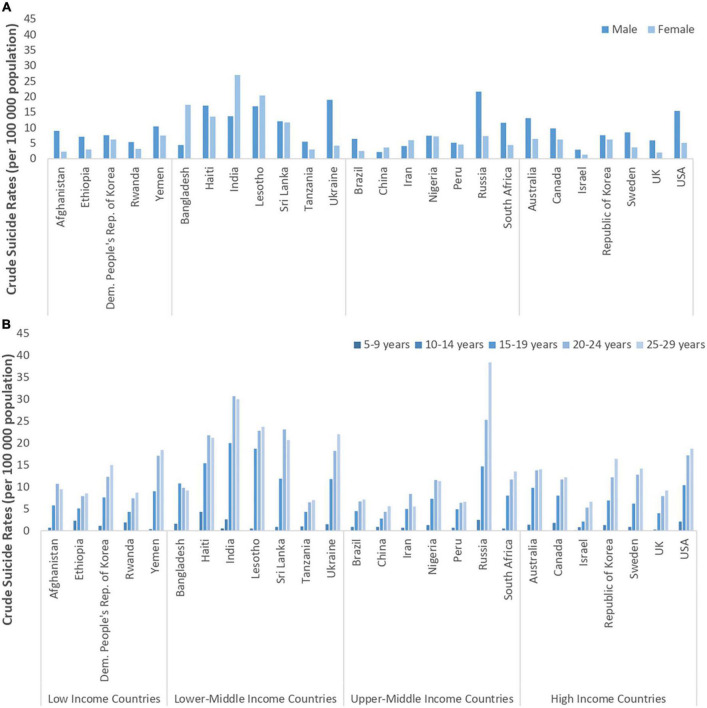
Source data: World Health Organization Global Health Observatory data repository (http://apps.who.int/gho/data/node.main.MHSUICIDE5YEAR AGEGROUPS?lang=en, 1a, 1b; http://apps.who.int/gho/data/node.main.MHSUICIDEAGEGROUPS15293049?lang=en, 1c) 2016 data; accessed October 24, 2019. **(A)** Crude suicide rates among 15–19 year-old children, by sex. Sex differences in the 15–19 age category vary between countries within across income categories. **(B)** Crude suicide rates among individuals 5–29 years, both sexes, by 5-year intervals. Suicide rates are consistently lowest in the 5–9 age range and increase through to age 24, but are heterogeneous around the world.

The global trend for decreasing suicide rates is also reflected among youth aged 15–29 (4), although suicide remains the fourth leading cause of death overall in this age group (11). Eighty-eight percent of adolescents who die by suicide live in LMICs (11). Although this rate is high, it reflects the proportion of adolescents worldwide living in LMICs (90%). A cross-national study of suicide rates among adolescents reported a global suicide rate of 3.77 per 100,000, with higher suicide mortality rates reported in older adolescents aged 15–19 years compared to younger adolescents aged 10–14 (6.04 vs. 0.93 per 100,000 population), and among males compared to females, all ages combined (4.91 vs. 1.99 per 100,000). Older adolescent males also had higher rates than younger adolescent males (8.41 vs. 0.76 per 100,000) (15). Of note, although higher rates of suicide deaths are observed among males in general, this gender-based trend is inverted for adolescents aged 15–19 years, with higher suicide rates reported among females than males, although these rates are approaching parity in recent years (4). These differences for older adolescents may be particularly impacted by the higher rates of suicide mortality in females 15–19 years old in China and India, two countries with large populations, inverting this trend across genders for older adolescents compared to other age groups (16). Figure 1 shows the variations in crude suicide rates for selected countries by gender (a.) and age group (b.) across World Bank Country Income Levels (9).

### Suicidal ideation and attempts

#### Global population

Although suicide mortality data is not always easy to obtain (2), efforts to harmonize practices have permitted between country comparisons for most age groups. In contrast, data on suicidal ideation and attempts is particularly difficult to obtain and compare between countries. This data is particularly lacking in LMICs due to obstacles such as poor registration of attempts, stigma and legal issues associated with suicide, and lack of health services. Nevertheless, existing data indicates that, as expected, suicidal ideation and suicide attempts are much more prevalent than suicide deaths. The WHO World Mental Health Surveys from 2001 to 2007 indicated that the average 12-month prevalence estimates in LMICs (all ages combined) are 2.1% for ideation and 0.4% for attempts, compared to 2.0 and 0.3% in HICs (3). Rates of suicide attempts are also higher among individuals who report previous suicidal ideation within the last 12 months in LMICs (20.2%) compared to HICs (15.1%) (3, 4). However, there is great heterogeneity in the prevalence of suicidal behaviors across LMICs (and HICs) and within particular subgroups of the population.

#### Adolescents and youth

Evidence suggests that suicidal ideation is most frequent among young people, with lifetime prevalence of 12.1–33%, and lifetime prevalence for suicide attempts of 4.1–9.3% (4, 5). Youth who attempt suicide have also increased one-year risk of repeated suicide attempts at 16.3%, and 1- and 5-year risk of suicide at 1.6 and 3.9%, respectively (6). In a WHO-led survey of adolescents (13–17 years) across 59 LMICs, the 1-year prevalence for suicidal ideation and suicide attempts were 16.9 and 17%, respectively (7). Of note, these reported rates for suicide attempts are higher than those typically reported for HICs (4, 8) calling for a reflection on potential different trajectories from suicidal ideation to behaviors in LMICs versus HICs (17). In addition, variations in the 12-month prevalence of suicidal ideation among adolescents have been reported across country income groups, with lowest rates in low-income countries (11.0 per 100,000 population) and highest rates among upper-middle-income countries (17.0 per 100,000 population), compared to rates of 13.0 in HICs (9). Differences also emerge based on age and gender, with older adolescents and girls having a higher prevalence of suicide attempts than their younger or male counterparts (17.6% for 15–17 years vs. 16.2% for 13–15 years; 17.4% for girls vs. 16.3% for boys), with similar trends observed for suicidal ideation (7). However, large heterogeneity in suicidal ideation and attempts is also observed among women aged 15 and older across LMICs. A WHO-led population-based study found a 4-fold variation in rates of lifetime suicidal ideation, a 7-fold variation in past month suicidal ideation, and a 15-fold variation in suicide attempts across LMICs (10). For example, prevalence of past-year suicidal ideation ranged from 1.9% (Serbia) to 13.6% (Peru); prevalence of lifetime suicidal ideation ranged from 7.2% (Tanzania) to 29% (Peru); and prevalence of lifetime suicide attempts ranged from 0.8% (Tanzania) to 12% (Peru) across the studied countries (10). Taken together, these reports demonstrate great heterogeneity in the prevalence of suicidal ideation and attempts across country-income levels, across countries, across youth age groups, and across genders.

## Factors contributing to suicide risk

**FIGURE 2:**
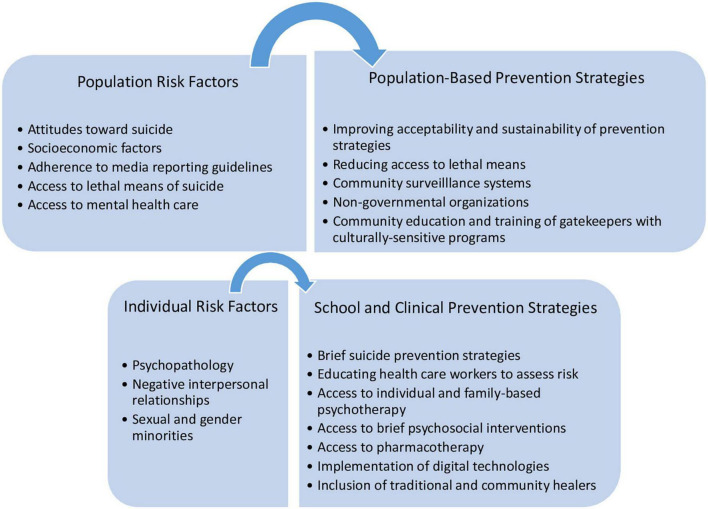
Youth-specific suicide risk factors and related prevention strategies.

Youth in LMICs, particularly older adolescent girls, exhibit high prevalence of suicidal ideation, attempts, and suicide mortality. The biopsychosocial model posits suicide risk as the result of the interplay between distal and proximal risk factors (18). This model provides a comprehensive framework to represent the diversity of factors that may contribute to suicide, including population factors and individual factors (18). In this section, we chose to highlight special considerations in some of the risk factors for suicide described in this model as they pertain to young people in LMICs (see Figure 2, for an overview).

### Population risk factors

At the population level, factors related to social structures, values, economic turmoil, or environmental factors affect suicide rates (18). Such population-wide contributors to suicide risk are frequently the object of public policy initiatives to prevent suicide.

#### Attitudes toward suicide

In 35 LMICs, specific laws (including Sharia law) and punishments exist to sanction suicide attempts, with penalties ranging from small fines to imprisonment (19, 20). The ongoing criminalization of suicide in these countries has been associated with higher suicide rates, particularly among women from countries with lower human development indices (i.e., lower life expectancy, lower education levels, and lower GNI) (21). The designation of suicide attempt as an offense may add to feelings of shame, guilt, or pre-existing distress of individuals who attempt suicide, including among youth, and may deter help-seeking and receiving immediate physical care for injuries related to suicide attempts (22), as emergency treatment is frequently delayed in view of legal aspects. The lack of help-seeking due to fear of negative judgment could contribute to the isolation and hopelessness of adolescents, which are important risk factors for suicidal behavior (23). Criminalization of suicide may also exacerbate the use of more lethal methods to escape prosecution (20), and lead to underutilization of mental health services and under-reporting of suicide and suicide attempts, which may skew the official statistics (18, 19).

#### Socioeconomic factors

In LMICs, poverty is widespread and may directly or indirectly affect suicide risk. Two systematic reviews on the association between poverty and suicidal behavior in LMICs reported an overall positive correlation between economic adversity and suicide and suicide attempts (24, 25). Moreover, countries with greater income inequality tend to have a larger ratio of male to female suicides among adolescents. However, individual economic adversity is more strongly associated with suicide-related outcomes than macroeconomic, national wealth indices (15). In addition to poverty, political conflict has led to the displacement of 25.9 million refugees, among which 67% have been hosted by LMICs (26). Most refugees in LMICs live in refugee camps with poor infrastructure and limited services, where the prevalence of suicide attempts ranges from 3 to 34% (26, 27). Lifetime prevalence of suicidal ideation among young migrants and refugees has also been estimated between 19 and 40% (27, 28). Among youth in these settings, the experience of interpersonal violence has been associated with the higher prevalence of suicidal ideation (28, 29). Therefore, chronic poverty, migration, and refugee status might contribute to suicide risk both distally, through long-term economic risk factors, and proximally, through personal economic adversity, displacement, and interpersonal violence, particularly among young people.

#### Adherence to media reporting guidelines

Portrayals of suicide in the media are a known contributor to suicidal ideation, suicide attempts, and suicide, particularly when the portrayal is romanticized or sensationalized (30). Media guidelines for reporting of suicide deaths have been established by the WHO (31), and irresponsible reporting and portrayal of suicide may contribute to the imitation of suicide attempts, particularly for youth who are highly susceptible to media influences (32). Further, Internet exposure to suicide-related content may negatively influence mental health, particularly in youth (33). LMICs may face numerous challenges to the implementation of media reporting guidelines, such as poorer health literacy, inadequate sensitization of media personnel regarding mental health issues, and lack of adequate legislation to enforce the implementation of these guidelines (34).

#### Access to lethal means of suicide

Patterns in the choice of suicide method observed across countries reflect sociocultural norms of the availability and perceived acceptability of methods (35). Among youth globally, the most frequently used method is hanging, followed by firearms among adolescent boys and poisoning by pesticides among adolescent girls (36). Pesticide poisoning is most common in LMICs, representing 13.7% of global suicides (37). The availability of pesticides in LMICs, particularly in China and India with large populations, could contribute to the higher rates of impulsive suicides among adolescent girls and the global importance of this suicide method (18). Self-immolation, which exists almost exclusively in LMICs (10–30 vs. 0.06–1% in HICs), is also a major cause of death and disability in parts of the Middle East and Central Asia, particularly among girls between the ages of 16 and 19 years old, recently married (within 2–5 years), and with minimal education or literacy (38, 39).

#### Access to mental health care

Systemic factors which affect mental health service accessibility also impact suicide risk (40). Individuals in LMICs, particularly those with severe mental health difficulties, have fewer 12-month contacts with any mental health services and less continuity of services compared to individuals in HICs (41). These exacerbated difficulties accessing health care and specialized services in LMICs have been associated with higher suicide rates in these areas (42). In a study of 58 LMICs, 67% showed a national shortage of psychiatrists, 95% a shortage of nurses, and 79% a shortage of psychosocial care providers (43). Poorer access to health services in LMICs is thought to interact bi-directionally with other individual-level risk factors thereby increasing suicide risk among adolescents (44). These shortages are attributed to many factors including discrimination and stigma against mental health patients, and lacking human and financial resources in mental health services, among others (44).

### Individual risk factors

In addition to population-level factors, individual risk factors stemming from clinical and psychosocial factors contribute to the accumulation of suicide risk in an individual (18).

#### Psychopathology

In HICs, there is a well-established link between psychopathology, particularly depression, and suicide risk. However, evidence suggests the prevalence of psychiatric disorders among adults with suicidal behavior in LMICs is lower than in HICs (45). This association is more poorly understood among youth in LMICs, but recent evidence shows that depressed adolescents in LMICs are 6.6 times more likely to attempt suicide than their non-depressed counterparts (46). Moreover, cigarette smoking, alcohol use, and anxiety, were significantly associated with suicide in adolescents from 40 LMICs (23, 47–49). These difficulties are also independently associated with increased aggression, impulsivity, and hopelessness, as well as poor sleep or insomnia, all of which also contribute to suicidal ideation and suicide attempts in this population (49, 50). In addition, early life adversity, irritability, cognitive difficulties, and certain personality traits are thought to play an important role in youth suicidal behavior via their cross-sectional and longitudinal associations with psychopathology (51–54). There thus seem to be differences in primary diagnostic predictors for suicide among youth in LMICs compared to HICs. Notably, cultural differences in perceptions and understanding of psychopathology, particularly of depression, may contribute to these differences in their prevalence, clinical manifestation, diagnosis, treatment response, and associations with suicidal behaviors across cultures (55).

#### Negative interpersonal relationships

Youth are particularly susceptible to social influences, and feelings of loneliness were the strongest individual risk factor among adolescents who made a suicide plan within 53 LMIC countries (56). Loneliness may result from various negative interpersonal situations that reduce youth’s sense of connection to family and friends. Problematic family relationships, such as negative parent-child interactions or lack of perceived parental support have also been associated with youth suicidal behaviors (57). Further, in a mental health survey of 21 HICs and LMICs, parental depression and anxiety disorders were found to predict onset and persistence of suicide plans among adolescents, whereas parental antisocial personality and anxiety disorders predicted suicide attempts among adolescent ideators (58). Parental death by suicide was also a strong predictor of suicide attempts among youth (58). Conversely, greater parental support and positive affect, as well as adaptive attachment representations are important factors associated with decreased suicide risk (59).

Social relationships with peers are also important in adolescence and may affect suicide risk. For example, a systematic review examining 27 school-, community-, and hospital-based studies of youth (12–25 years) in LMICs found that having friends was a significant protective factor against suicidal behaviors (60). In turn, a major contributor to suicidal ideation and suicide attempts in youth is peer victimization. An investigation of 134,229 adolescents aged 12–15 in LMICs found that bullying victimization was specifically associated with threefold risk of suicide attempts in 47 of the 48 countries investigated, and that this association seems dose-dependent, with the 12-month prevalence of suicide attempts averaging 5.9% if not bullied and increasing fivefold to 32.7% if bullied 20–30 days per month (61). Moreover, evidence suggests that female adolescent perpetrators of bullying and interpersonal violence are 2.7 times more likely to report suicidal ideation than non-perpetrating girls (62). Thus, victimization and perpetration of interpersonal violence among peers are important to consider in evaluating suicide risk among adolescents, particularly girls, in LMICs.

Indeed, young women in LMICs are particularly vulnerable to the experience of interpersonal violence. Population-based studies in LMICs have shown that adolescents and young women who ever experienced emotional, physical, or sexual violence by a caregiver or an intimate partner were 1.4–9.18 times more likely to report lifetime suicidal thoughts, and 3.8 times more likely to ever attempt suicide (63–65). The prevalence of suicidal ideation among female victims of intimate partner violence ranges from 28% in the Philippines, 48% in Brazil, and over 60% in Egypt and India (65). Additionally, there is a dose-response effect where the proportion of women reporting suicidal thoughts increased from just 1% if exposed to no form of violence to 15–16% when exposed to physical, sexual, and psychological abuse (62). Women from LMICs in South Asia are also particularly vulnerable as they may experience forced or arranged marriage, young age of marriage, and denial of their choice of spouse (64). Related issues such as infertility, pressure to have children, especially male children, difficulties providing a dowry, and troubled relationships within the family, are also associated with suicidal behavior (66). Together, these findings suggest that having supportive and positive relationships with family and friends are important to reduce suicide risk, yet certain individuals may experience significant barriers to achieving this support.

#### Sexual and gender minorities

Limited research has explored suicide risk among sexual and gender minorities in LMICs. From research conducted in HICs, youth that belong to a sexual minority were twice as likely to attempt suicide than those who did not belong to such groups (67). One study among adolescents identifying to sexual minority groups in China found that adolescents with same-sex and both-sex attractions had higher prevalence of past-year suicidal ideation and suicide attempts than their heterosexual counterparts (68). Although sexual minority groups face a variety of social and legal challenges in all countries, in LMICs these challenges may be compounded by discrimination, which can exacerbate suicidal ideation and attempts in this vulnerable population (69). Additional research among sexual and gender minority groups in LMICs, particularly among youth is warranted (69, 70).

## Population-based prevention strategies

From a public health perspective, suicide prevention strategies can be implemented at the population- level, or can be focused on particular demographic groups or at-risk individuals. Combination approaches in health care systems have already helped reduce suicide in several countries even if the evaluation of the benefit attributable to each component is still needed (71). Intervention targets may include three main groups of actors involved in the field of youth suicide: (1) policy makers/government officials; (2) clinical and school settings; and (3) researchers.

### Policy makers and government officials

Policy change or interventions in schools, workplaces, specific communities, or in individual health care services are helpful to reduce suicide (18, 72, 73). In line with recent interventions in China to reduce depression among Chinese youth, effective youth suicide prevention strategies in LMICs should be tailored to the specific needs or constraints of target populations based on developmental factors and prior cross-cultural adaptation of prevention and intervention strategies reflecting the local availability of services and material support as well as the local culture, traditions, and representations of mental disorders (74). Effective cross-cultural adaptation is an ongoing interaction between the intervention and the adopters’ values, norms, and perceived needs. Medical sociology and anthropology might be useful at fostering task-shifting initiatives between mental health providers and community care providers to improve pathways to suicide prevention. This process could improve the acceptability and sustainability of prevention strategies resulting from HIC experiences. Nonetheless, LMICs face significant barriers to the prioritization of such programs. In these countries, other public health concerns (e.g., infectious diseases, malnutrition, infant, and maternal mortality) receive large portions of the limited resources available, and suicide prevention is often a lower priority (75). Only 10% of low and low-middle-income countries have a standalone, government-adopted suicide prevention strategy (76). Moreover, the available mental health workforce for 100,000 population is 1.6 in low-income countries, 6.2 in lower middle-income countries, and 20.6 in upper middle-income countries, compared to 71.7 in HICs. The limited human and economic resources, along with the wide variability of suicide frequency in LMICs suggest that locally relevant, culturally appropriate, and cost-effective interventions for suicide prevention are needed. Table 1, adapted from the Disease Control Priorities (third edition) (12) describes potential suicide prevention strategies in LMICs using a stepped-care model considering the risk factors described above at the population- and individual levels.

**TABLE 1:** Suicide prevention and intervention strategies in LMICs.

Population-wide strategies
● Restrict the availability of toxic pesticides and other commonly used methods
● Decriminalize suicide
● Reduce the availability and excessive use of alcohol and illicit drugs
● Work with national and local media organizations to limit inappropriate reporting of suicides
● Conduct campaigns to reduce the stigma associated with suicide and mental disorders and to encourage help-seeking behavior
● Provide adequate economic and welfare support to individuals who are unemployed, disabled, or destitute
Community-based strategies
● Non-Governmental Organizations: provide suicide hotlines and crisis centers, and promote social cohesion and interpersonal support in communities and families
● Initiate school-based mental health promotion programs to enhance psychological resilience, problem-solving skills, and appropriate help-seeking behavior
● Organize community-based safe storage activities for pesticides, other poisons, and medications
● Provide gatekeeper training to teachers, people looking after refugees, police, social workers, practitioners of alternative systems of medicine, traditional healers, and other individuals who interact with suicidal individuals
● Implement community-wide health promotion programs to encourage help-seeking for psychological problems and reduce alcohol and drug abuse, child abuse, and domestic violence
● Implementation of community care and Zero suicide initiatives
Health and social care strategies
*Prevention and care for persons and families affected by mental, neurological, and substance disorders*
● Conduct brief interventions for people who have attempted suicide
● Train primary health care workers in the identification and management of individuals at high risk of suicidal behavior
● Improve health care professionals’ identification and treatment of depression and alcohol or drug abuse
● Provide regular follow-up, social support, and (if appropriate) cognitive behavioral therapy or other psychological treatment to individuals who have attempted suicide
● Improve the medical management of poisoning with pesticides and other poisons associated with high case-fatality
● Establish services to support individuals bereaved by suicide (post-vention services)

Adapted with permission from Disease Control Priorities 3 (12).

At the population level, reducing access to lethal means of suicide can reduce the potential for impulsive suicides (77). It is possible that the easy availability of pesticides could drive the higher suicide mortality among young women in LMICs compared to HICs (4, 14). Measures proposed to prevent suicide by pesticide poisoning include targeting the accessibility of these substances by: ratifying, implementing and enforcing relevant international conventions on hazardous chemicals and wastes; bringing in legislation to remove locally problematic pesticides from agricultural practice; enforcing regulations on the sale of pesticides; reducing access to pesticides through safer storage and disposal by individuals or communities; and reducing the toxicity of pesticides (78, 79).

Given the limited availability and access to services in LMICs, suicide prevention is more often a social and public health objective than a traditional exercise in the mental health sector. Moreover, the misclassification of deaths and limited accessibility to emergency health services disproportionately affect young women in LMICs (80). Where the registration system is lacking, more effective community surveillance systems are essential to better understand patterns of suicide attempts across different LMIC settings (81, 82). This approach harnesses community action through building community capacity, while pragmatically recognizing the finite health resources in LMICs. Community-level strategies that may have potential in preventing suicide include using the services of non-governmental organizations, community education about self-immolation, and training of individuals to recognize symptoms of distress (gatekeepers) with culturally sensitive programs (83). There is, however, a need for more extensive studies to improve the evidence base for these suggestions in LMICs before they can be recommended for specific contexts.

### Clinical and school settings

At the level of individual health care and social services, evidence suggests that cost-effective, brief suicide prevention strategies are both feasible and effective in LMICs despite human and economic obstacles. For example, a WHO-led, multisite intervention study on suicidal behavior conducted in five LMICs (Brazil, India, Sri Lanka, Iran, and China) demonstrated that brief interventions and ongoing supportive contact with suicide attempters was associated with fewer deaths from suicide during the 18-month follow-up compared to a treatment as usual group (84). Postcard-based interventions to reduce suicidal ideation and suicide attempts following hospitalization have also shown positive results (85). Another key tool for suicide prevention is the education and training of health workers to ensure that psychosocial support is provided to those in need. There is a growing number of LMICs where suicide awareness and skills training have been implemented in primary care services. Educating health care workers to recognize culturally relevant symptoms of depression, anxiety, and other mental and substance use disorders in primary care and emergency department settings and to assess imminent risk of suicide are important for determining the level of care and referral for treatment and reduce suicide risk (86). This can be implemented through the WHO mhGAP Intervention Guide in non-specialized health settings (87). Training should take place continuously or repeatedly over years and should involve the majority of health workers in a region or country. It is important to consider and tailor the programs to local risk factors to be successful within countries and across cultures.

A specific area of continued concern in youth suicide prevention relates to adolescent depression and impulsivity disorders, and access to specific treatments. Evidence is accumulating for the utility of interventions acknowledging the complex interplay between interpersonal functioning, particularly family functioning, and suicidal ideation and behaviors among this demographic, although findings remain mixed (88). The effectiveness of longer-term individual and family-based psychotherapeutic approaches in clinical settings has not been as well studied in youth as in adults and is not yet well established, despite some promising results (e.g., cognitive-behavior therapy, dialectical-behavior therapy) (89, 90). Nonetheless, evidence for psycho-pharmaceutical and brief psychosocial and psychological interventions is growing. Across age groups and including adolescence, access to antidepressants is associated with decreased rates of suicide (91). Short-term physician prescriptions of potentially lethal medication limiting the number of pills available (especially for tricyclic antidepressants) is an important way to decrease impulsive suicide attempt and suicide in youth. Furthermore, whereas adolescents have a high prevalence of lifetime contact with services for emotional or substance-related difficulties, fewer than 20% use services within 1 year of suicide or suicide attempt suggesting greater obstacles to accessing these specialized healthcare services (40). Brief interventions in emergency departments or during psychiatric hospitalizations for suicidal youth are also important to improve access to mental health care and prevent future suicide attempts (92). One option for improving care is through the systematic implementation of digital technologies in the monitoring and care of mental health patients. In many countries, electronic devices are omnipresent and offer an opportunity for innovative online interventions, which are just beginning to be explored. The use of new technologies in service delivery has the potential to overcome barriers to accessibility and improve outcomes monitoring, and could lead to greater cost-efficiency of care (93) [e.g., iBobbly app (94), or the As Safe as Possible App (95)].

In addition to clinical interventions, evidence-based approaches include the prevention of childhood maltreatment and interpersonal violence, as well as family interventions aimed at promoting positive parenting, parent-child interactions, and lessening parental psychopathology (96, 97). School-based interventions are also effective to reduce suicidal ideation and suicide attempts (98). Some have targeted young children from vulnerable social environments through teachers’ implementation of pro-social skills [e.g., the Good Behavior Game (99)]. Other programs aimed to identify at-risk youth through training of gatekeepers, and some have been designed to reduce stigma about mental illness and to augment help seeking. Other programs have also combined different strategies, including improved mental health knowledge (curriculum-based intervention), help seeking, and better coping with depression and suicidal behavior (100, 101). Although promising, clear and convincing data comparing programs, delivery methods, and short- and long-term outcomes using rigorous methodologies are required to translate such initiatives into public policy in LMICs. The utilization of community health workers and other community gatekeepers, including traditional healers, in the delivery of care, also need to be assessed. Both traditional healers and biomedical mental health providers could benefit from a combination of both practices despite differing conceptualizations of mental illness causation (102, 103).

### Researchers

Suicidal ideation and attempts are challenging to identify and effectively prevent due to ongoing stigmatization in many countries and the many risk factors for these behaviors. In this review we highlight key risk factors to consider when evaluating suicide risk in youth in LMICS, and highlight some individual- and population-level measures that could be pursued among these groups to prevent suicide. More research is needed on the local determinants of suicide and on the acceptability of programs that have been shown to be effective in HICs, including public policy changes and long-term and short-term clinical and psychosocial interventions. Both material and technical support for these studies through collaboration between research teams accustomed to evaluating suicide prevention efforts in HICs and local research teams in LMICS would help improve knowledge of optimal suicide prevention strategies in these settings.

## Conclusion

Youth from LMICs represent a population at high risk for suicidal ideation, attempts, and death.

Through continued investments in research on suicide, development of effective treatments, and implementation of comprehensive prevention strategies, suicide rates have begun to drop in some countries. Even in countries where rates have been declining, there are concerns that youth from LMICs continue to carry a high number of suicide deaths. In its Global Action Plan, the WHO identifies sustainable development goals for non-communicable diseases, among which one is to reduce suicide mortality by one third between 2015 and 2030 (104). Despite the poor available data, if current trends continue, only 3% of 118 countries will attain this target, highlighting the need for countries around the world to improve their data on suicide, as well as to significantly reduce suicide rates by implementing multimodal prevention strategies. No single approach or treatment is likely to be effective in preventing suicide due to the variability in demographic, clinical, and cultural presentations of suicidal ideation and behaviors. Thus, multi-level interventions are needed. At the population level, reducing access to lethal means such as pesticides for girls or firearms for boys in LMICs could be priority targets for prevention strategies given the level of evidence regarding their effectiveness in reducing suicide rates. At the individual level, there is a need for bolder policies facilitating access to effective care for mental disorders in LMICs, particularly for youths with mood and substance use disorders. Culturally-adapted and brief community psychosocial interventions as well as contact with trained mental health care providers could have a significant impact especially for the most vulnerable youths. Development of innovative online interventions should be quickly explored and prioritized. Although strategies may be difficult to implement in LMICs, where resources are scarcer and access to trained healthcare providers and services may pose unique challenges, suicide prevention in these countries should be a high priority.

## Author contributions

JR and SM conceived and developed an initial draft manuscript in consultation with DB, LV, SD, and GT, who regularly provided extensive feedback. MS assisted with reviewing and finalizing all aspects of the manuscript. All authors contributed to the final version of the manuscript.
